# An alternative solution for patient with high risk of coronary obstruction underwent TAVI procedure using a novel second-generation device – a case series

**DOI:** 10.1186/s13019-019-0859-1

**Published:** 2019-02-28

**Authors:** Hong Qian, Yucheng Chen, Zhaoyun Cheng, Yingqiang Guo

**Affiliations:** 1 Department of Cardiovascular Surgery, West China Hospital, Sichuan University, Chengdu, Sichuan 610041 People’s Republic of China; 2 Department of Cardiology, West China hospital, Sichuan University, Chengdu, Sichuan People’s Republic of China; 3 Department of Cardiovascular Surgery, Heart Center of Henan Provincial Peoples Hospital and Fuwai central hospital, Zhengzhou, Henan People’s Republic of China

**Keywords:** Transcatheter aortic valve implantation, Coronary obstruction

## Abstract

**Background:**

Obstruction of the left or right coronary artery is a rare but lethal complication during transcatheter aortic valve implantation (TAVI). The new J-Valve™ prosthesis is a new second generation TAVI device which has several features to avoid the coronary obstruction such as low profile design and clip fixation of the native leaflets. The aim of this study is to report our initial experience of using this valve in treating patient with high risk factors for coronary obstruction during TAVI procedure.

**Case presentation:**

Three high surgical risk patients (All females with 77, 76, and 75 years old) with symptomatic aortic stenosis were enrolled. All patients have the common feature of low coronary ostium height (< 10 mm) with narrowed aortic sinus (< 30 mm) on CT angiogram and marked leaflet calcification. Three 25 mm J-Valve prostheses were successfully implanted through trans-apical approach. No coronary obstruction was noted for these patients. Effective aortic open area was significantly increased after valve implantation (Preoperative 0.7, 0.7 and 0.65 cm^2^ – Postoperative 1.8, 1.9 and 2.0 cm^2^). Only one patient was noted to have trivial degree paravalvular leakage.

**Conclusion:**

The new J-Valve prosthesis is a new second generation TAVI device. This system may provide another safety treatment option for patient with high risk factor for coronary obstruction underwent TAVI procedure.

## Background

Transcatheter aortic valve implantation (TAVI) has evolved as a routine procedure to treat selected high-risk patients with severe aortic stenosis. Although obstruction of the left or right coronary ostium is a relatively rare but lethal complication during TAVI procedure, it is generally accepted that a distance between the coronary ostium and the annulus less than 10 mm or sinus of Valsalva diameter below 30 mm, and existing of bulky calcified leaflet are risks for coronary occlusion as for several type of classic TAVI devices such as Edward SAPIEN and Medtronic CoreValve [1, 2]. The J-Valve™ system is a brand new second-generation TAVI device featured by three U-shape anatomically oriented devices “claspers” which could facilitate intuitive “self-positioning” valve implantation [3]. Due to the ability of embracing the native calcified leaflets during valve implantation and specially designed bear area on valve stent, this low-profile designed valve is especially suitable for patient who is at high risk for coronary obstruction. We then reported our initial experience of using J-Valve in treating patient with high risk factor for coronary obstruction during TAVI procedure.

## Case presentation

Three high surgical risk patients with symptomatic aortic stenosis who are at high risk for coronary occlusion during TAVI procedure were enrolled (three female patients). The basic characters shown in Table 1. These three patients have the common feature of low coronary ostium height (< 10 mm) with narrowed aortic sinus (< 30 mm). The effective aortic valve opening area (EOA) was 0.7 cm^2^, 0.7 cm^2^ and 0.65 cm^2^ in each patient with marked leaflet calcification. Aortic valve annular size was 24 mm, 25 mm, 24 mm on CT image.

**Table 1:** Basic character of three patients

Patient	Log-Euroscore I(%)	Aortic Valve	Calcification	LCH	RCH	Aortic Sinus	Annular size	Valve Size
No1 77y Female	21%	Severe AS	Moderate	7.8 mm	7.8 mm	27 mm	24 mm	25 mm
No2 76y Female	20%	Severe AS	Severe	8.9 mm	9.5 mm	29 mm	25 mm	25 mm
No3 75y Female	22%	Severe AS	Bulky	8.2 mm	7.9 mm	27 mm	24 mm	25 mm

*LCH* Left coronary ostium height, *RCH* Right coronary ostium height

**Fig. 1:**
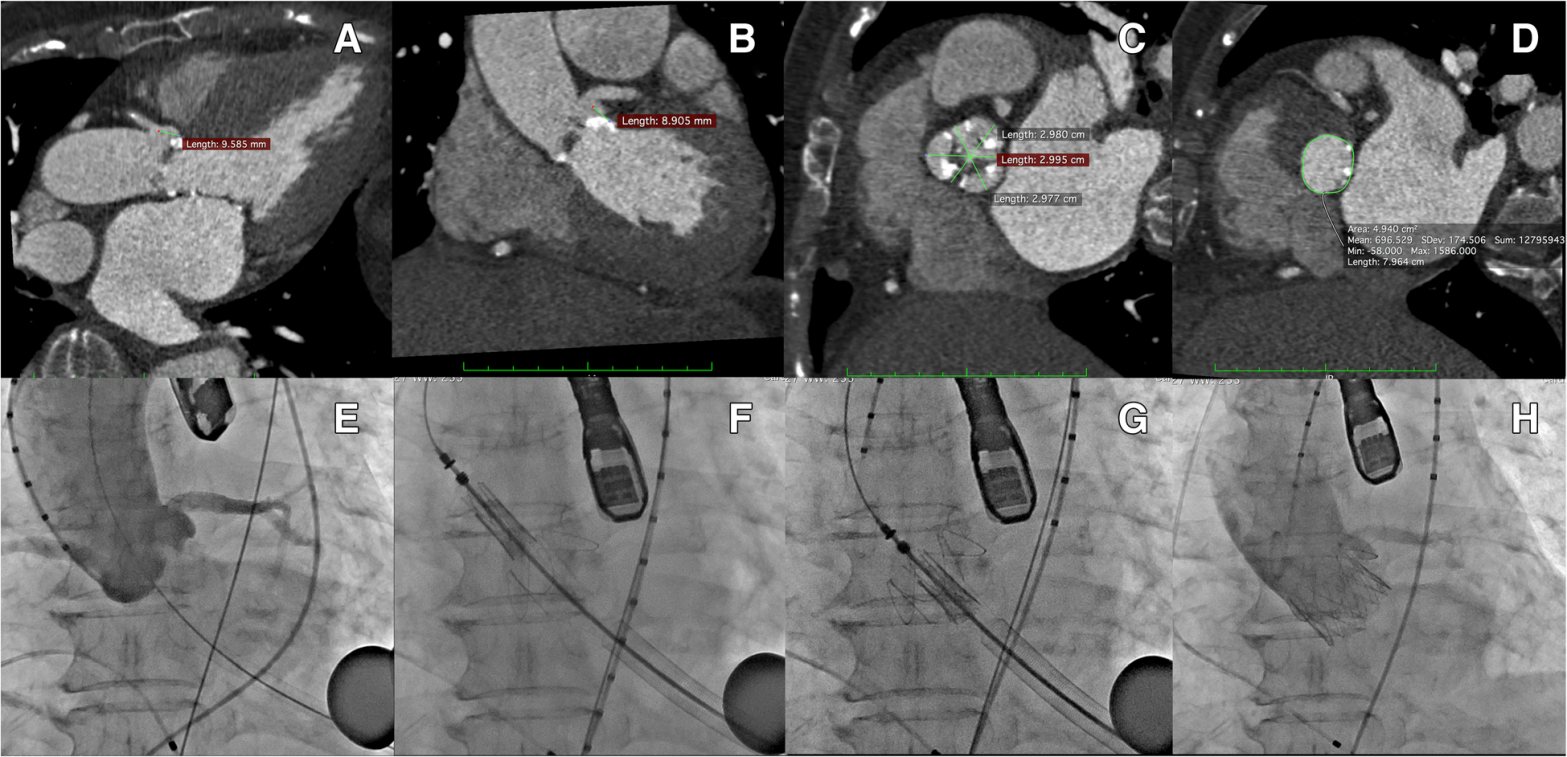
J-Valve implantation in a patient with high risk of coronary obstruction. Upper Panel: Note both left and right coronary height was all less than 10 mm with narrow aortic sinus (**a**-**d**). Lower Panel: Angiogram also revealed low coronary ostium height (**e**). Valve deployment process (**f**-**h**): Three Clasper were released and pushed back gently into the aortic sinuses, the valve was retrieved into the annular plan with the help of the locking device. The valve was then deployed without rapid ventricular pacing, further angiogram confirmed good position of the valve while without obvious paravalvular leakage

The TAVI procedure using J-Valve™ prosthesis was performed through trans-apical approach (Fig. 1). The procedure detail is described previously [3]. Briefly the apical puncture was done with a super-stiff guide-wire placed into the descending aorta. A 14F sheath was carefully introduced into the left ventricle followed by a balloon catheter. Pre-procedural balloon-valvuloplasty of the native valve was performed under rapid pacing. In parallel, a J-Valve™ prosthesis was crimped into the 27-F Ausper-AS delivery system (Three 25 mm prostheses were used). The delivery system was bluntly inserted into the left ventricle and advanced under fluoroscopic guidance into a supra-annular position. Three ‘U-shaped’ graspers were then completely released first and carefully placed into the corresponding aortic sinus by gently pulling back the delivery system, thereby embracing the native leaflets. The angiogram was then performed to confirm that all the graspers were positioned correctly into the each aortic sinus. The valve was retrieved back gently into the annular plan with the guidance of the graspers and deployed without rapid ventricular pacing. After wound closure, the patient was transferred to the intensive care unit. Further postoperative course was uneventful. At discharge, TTE revealed an excellent valve function with EOA (1.8 cm^2^, 1.9 cm^2^, 2.0 cm^2^ in each patient) without any relevant incompetence. One patient was noted to have trivial degree paravalvular leakage.

## Discussion

Although the incidence of coronary obstruction reveled in large TAVI series and registries are relatively low, it is still the most lethal complication of TAVI procedure [1]. Evidences have shown that a distance between the coronary ostium and the annulus less than 10 mm or sinus of Valsalva diameter below 30 mm, and existing of bulky calcified leaflet are risks for coronary occlusion as for classic TAVI device such as Edward SAPIEN and Medtronic CoreValve due to their design [2]. Contrast to western population, Asian patients always associated with low body weight, relatively small aortic root and low coronary orifice height. In some specific situation, choose of the classic TAVI device may warrant caution or even contraindicated.

**Fig. 2:**
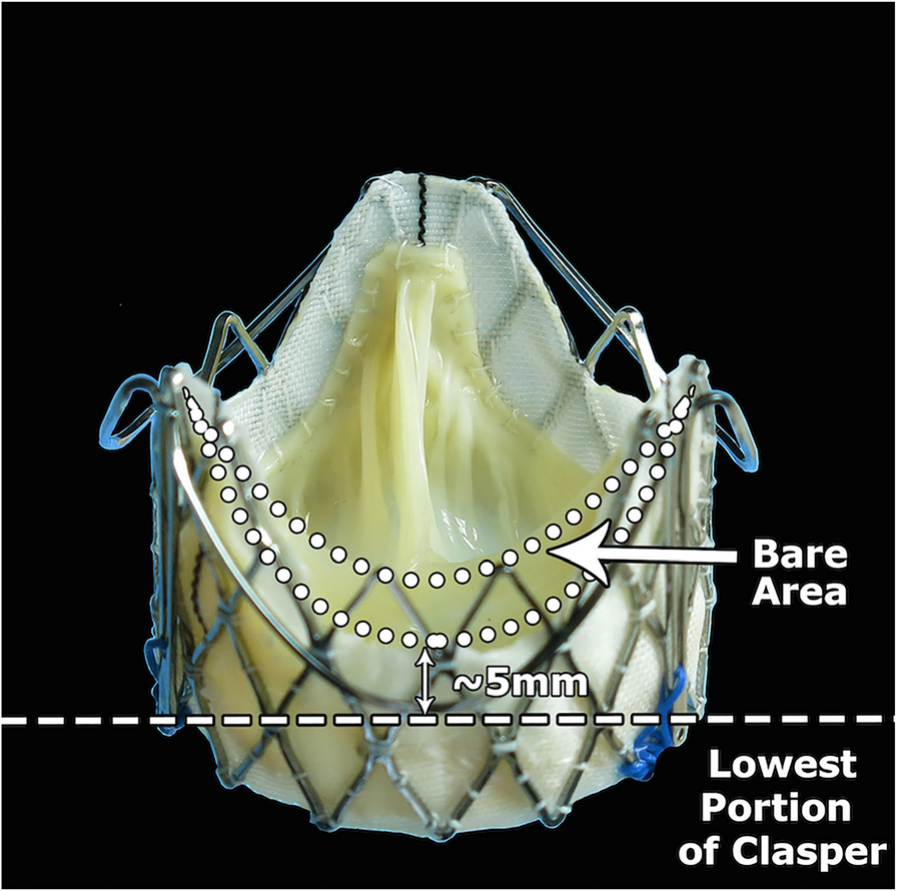
The J-Valve™ system is a new second-generation TAVI device that is featured by three U-shape anatomical orientated devices-“Clasper” encircling around the valve frame. It has a low-profile design with extra bare mental area in coronary orifice region to minimize the risk of coronary obstruction

The J-Valve™ system is a new second-generation self-expendable low-profile prosthesis that is featured by three U-shape anatomical orientated devices — “Clasper” encircling around the valve frame. The design concept of this device is to facilitate intuitive ‘self-positioning’ valve implantation through embracing the native aortic valve leaflet. In this case serious, successful TAVI procedure was done using this unique system in all three patients who are at high risk for coronary obstruction with low coronary height and narrowed aortic sinus. This valve have several unique features to minimized the risk coronary obstruction including 1) More accurate position of the device with anatomical orientated devices to avoid malposition; 2) Low-profile design with extra bare mental area in coronary orifice region (Fig. 2); 3) Unlike the traditional design of classic stent valve system, the unique designed U-shape claspers could embrace the native aortic valve leaflet (clip mechanism) then subsequently leaving the coronary ostia freely accessible from heavily calcified aortic leaflet; 4) Meanwhile, J-Valve™ system has the advantage of achieving better anchoring through embrace the leaflets, then diminish the necessity of oversizing and minimize the risk of coronary obstruction.

## Conclusion

The new J-Valve™ prosthesis is a brand new second generation TAVI device. This system may provide a safety treatment option for patient with high risk factor for coronary obstruction underwent TAVI procedure.

## Abbreviations

EOA: Effective aortic valve opening area
TAVI: Transcatheter aortic valve implantation

## References

[1] Ribeiro HB, Webb JG, Makkar RR (2013). Predictive factors, management, and clinical outcomes of coronary obstruction following transcatheter aortic valve implantation: insights from a large multicenter registry. JACC Cardiovasc Interv.

[2] Ribeiro HB, Nombela-Franco L, Urena M (2013). Coronary obstruction following transcatheter aortic valve implantation: a systematic review. JACC Cardiovasc Interv.

[3] Zhu D, Wei L, Cheung A (2016). Treatment of Pure Aortic Regurgitation Using a Second-Generation Transcatheter Aortic Valve Implantation System. J Am Coll Cardiol.

